# Unravelling the Relationship between Animal Growth and Immune Response during Micro-Parasitic Infections

**DOI:** 10.1371/journal.pone.0007508

**Published:** 2009-10-19

**Authors:** Andrea B. Doeschl-Wilson, Will Brindle, Gerry Emmans, Ilias Kyriazakis

**Affiliations:** 1 Sustainable Livestock Systems, Scottish Agricultural College, Edinburgh, United Kingdom; 2 Animal Health, Scottish Agricultural College, Edinburgh, United Kingdom; 3 School of Agriculture, Food and Rural Development, Newcastle University, Newcastle upon Tyne, United Kingdom; 4 Faculty of Veterinary Medicine, University of Thessaly, Karditsa, Greece; University of Liverpool, United Kingdom

## Abstract

**Background:**

Both host genetic potentials for growth and disease resistance, as well as nutrition are known to affect responses of individuals challenged with micro-parasites, but their interactive effects are difficult to predict from experimental studies alone.

**Methodology/Principal Findings:**

Here, a mathematical model is proposed to explore the hypothesis that a host's response to pathogen challenge largely depends on the interaction between a host's genetic capacities for growth or disease resistance and the nutritional environment. As might be expected, the model predicts that if nutritional availability is high, hosts with higher growth capacities will also grow faster under micro-parasitic challenge, and more resistant animals will exhibit a more effective immune response. Growth capacity has little effect on immune response and resistance capacity has little effect on achieved growth. However, the influence of host genetics on phenotypic performance changes drastically if nutrient availability is scarce. In this case achieved growth and immune response depend simultaneously on both capacities for growth and disease resistance. A higher growth capacity (achieved e.g. through genetic selection) would be detrimental for the animal's ability to cope with pathogens and greater resistance may reduce growth in the short-term.

**Significance:**

Our model can thus explain contradicting outcomes of genetic selection observed in experimental studies and provides the necessary biological background for understanding the influence of selection and/or changes in the nutritional environment on phenotypic growth and immune response.

## Introduction

Models that predict phenotypic responses from the interaction between animal genotypes and the environment are desirable both in the context of agricultural systems [1] and evolutionary ecology [2], [3]. Whereas extensive knowledge exists about the effects of animal genotype and nutrition on performance in infection-free environments [1], [4]–[8], understanding of the interactive influence of a host's capacities for growth and disease resistance and of nutrition on phenotypic responses under pathogen challenge is relatively limited.

There is plenty of evidence that nutrient availability can affect the ability of a host to control invading pathogens [9]–[11]. The effect can be attributed to increased nutritional costs associated with the development of an effective immune response [2], [12]. A problem arises if nutrient availability is scarce, in which case a trade-off between mounting an immune response and other body functions (e.g. growth, reproduction) occurs. Trade-offs are central concepts in evolutionary biology [13], [14], and a large body of studies has either indicated trade-offs occurring frequently amongst natural and semi-natural populations [15]–[17], or considered them theoretically [3], [18], [19]. Such populations are frequently confronted with trade-offs due to variation in food resource availability. By contrast, domestic livestock raised in controlled, nutrient rich environments are expected to face the dilemma of appropriate partitioning of nutrients to a lesser extent. However, the situation of sufficient nutrient supply may be compromised when domestic livestock are exposed to pathogens, as a common by-product of infection is a reduction in voluntary food intake, henceforth called anorexia [20]. While there is still speculation about the biological mechanisms underlying anorexia, it is well established that infection induced anorexia occurs across a wide range of pathogen and host species in both natural and domestic populations, and that this causes a trade-off between the immune response and other body functions [10], [21], [22].

As with the role of nutrition, it is well established that host genetics strongly influence how animals allocate nutrients [23]–[25] and thus how they respond to pathogen challenges. The ‘resource allocation theory’ of Beilharz et al. [26] states that ‘when environmental resources are limiting, all major components (e.g. growth, immunity, reproduction) of fitness are selected toward intermediate optimal values’. The theory implies that resource allocation preferences are genetically determined and that such preferences will be tailored to suit the environment in which selection is made. For domestic livestock, changes in host genetics, which are aimed at maximising fitness in natural populations, are often defined by the breeding goal, which primarily targets production traits [27]. Both experimental and simulation approaches have shown that artificial selection for production traits influences the performance-resistance relationship [27], [28], but the outcomes are often contradictory and the underlying mechanisms not fully understood (as reviewed by [27]). Similar conflicting outcomes arise from artificial selection for disease resistance, with some studies reporting improved performance [29], [30], while others report a performance decrease [31], [32]. Previous simulation studies of selection experiments suggest that the relationship between production and immune traits in a population depends strongly on the physiological status of the animals at the time of selection as well as on the genetic relationship (e.g. pleiotropy or linkage) between production and resistance traits [33], [34]. However, these studies have assumed that the performance-resistance relationship is mainly determined by host genetics and the infectious challenge, whereas the potential influence of the nutritional environment has been ignored.

In this study, a different approach to previous theoretical studies is adopted by exploring the hypothesis that the conflicting outcomes of selection experiments may arise through the nutritional environment of the host affecting the relationship between the genetic traits associated with growth and immune response. The underlying assumption of this study is that the host's genetic capacities for growth and immune response determine the animal's nutrient requirements and preference of allocating nutrients to either process, and that nutrient availability stipulates the extent at which the genetic potentials are expressed if the animal is under pathogen challenge. A similar approach had been adopted to model the interactive effect of host genotype and nutrition on gastro-intestinal parasitism in growing lambs [35], [36]. The model developed in this study focuses on micro-parasitic infections, which are characterised by short generation times and high rates of reproduction within the host [37].

## Methods

### (a) Assumptions

We consider only two resource demanding biological processes of a host: growth and immune response to pathogen challenge. Resource requirements for all other processes (e.g. maintenance, reproduction, coping with environmental stressors other than pathogen challenges, damage from within-host parasite replication) are assumed to be either negligible (e.g. the animal is assumed to be in a non-reproductive state and that requirements for the development of the reproductive apparatus are encompassed by those for growth) or fully satisfied during the time periods considered here. We further assume that the host has genetically intrinsic capacities for growth and immune response [25], [38] hereafter simply called genetic growth and resistance potential, respectively. Both these biological processes are associated with nutritional costs [10], [12].

Pathogen induced anorexia, i.e. a reduction in the host voluntary food intake, is considered to be the main reason that challenged animals cannot fully cover the nutritional requirements to achieve the above genetic capacities during infection and are required to distribute scarce resources between growth and the immune response [21], [26].

### (b) Model description

#### Genetically controlled capacity for growth and immune response

We assume that during the typical short time scales of micro-parasitic infection, the animal's intrinsic capacity for change in body weight (*dW_p_/dt*) is constant, i.e.
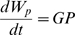
(1)where *GP* describes the animal's genetic growth potential in the absence of infection.

The host's genetically determined capacity of the immune response and associated within-host parasite dynamics are described according to the equations of Antia et al. [39], which have two variables, i.e. the (potential) intensity of the immune response (I_p_) and the parasite density (P), which interact according to
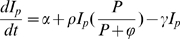
(2a)

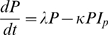
(2b)where *λ* is the replication rate of the parasite, *κ* is the constant rate for the elimination of the pathogen by the host's immune response, *α* and *γ* are the replacement and death rate of the immune cells, respectively, *ρ* is the maximum per capita replication rate of the immune response and φ represents the parasite density at which the rate of growth of immunity is half maximal.

#### Incorporating nutrition

Nutrient requirements are defined as the requirement for the most limiting nutrient in the food, which is assumed to be of the same kind for growth and immunity. We assume that nutrient requirements for growth (N_G_^*^) and immune response (N_I_^*^) are proportional to the resource demanding components of these processes. This is consistent with the principles of allocation of scarce resources adopted in many nutritional models [10], [35], [40] According to equations (1) and (2a) N_G_^*^ and N_I_^*^ are thus given by
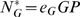
(3a)

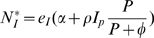
(3b)where the values of the efficiency parameters *e_I_* and *e_G_* represent the resource cost (in units of the most limiting nutrient) per unit increase in *I* and *W* respectively.

Infection induced anorexia is included in the model through a reduction in the desired nutrient intake - which is defined as the total amounts of nutrients required to satisfy the genetic growth and resistance potentials N* = N_G_^*^+N_I_^*^ - by a factor *r*. For simplicity and using empirical evidence, it is assumed that the degree of anorexia is proportional to the pathogen load (with proportionality constant *ν*) for small to moderate levels of infection [41], [42], and does not fall below a minimum *r*_min_ for a wide range of P values [21], [43]. Hence
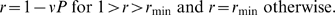
We assume that the host distributes available nutrients (*N*) between immunity (*N_I_*) and growth (*N_G_*) according to
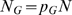
(4a)

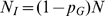
(4b)where
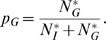
(5)This partitioning rule entails that the nutrient supplies for growth and immunity are reduced by the same proportion relative to their respective requirements if insufficient nutrients are available.

#### Actual growth and within-host parasite interaction

Combining equations 1, 3a & 4a, and equations 2, 3b & 4b, respectively, leads to the following expressions for the predicted actual growth and change in immune response in terms of allocated nutrients:
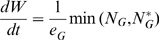
(6a)

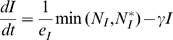
(6b)The change in pathogen load (*dP/dt*) is calculated according to equation (2b) after replacing *I_p_* with *I*:
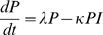
(6c)Equations 6a, 6b imply that actual growth and immune response equal their corresponding genetic potentials if sufficient nutrients are available, but are reduced by equal proportion if nutrients are scarce. Also note that the model for the within-host parasite dynamics (equations 6b, 6c) coincides with that of Antia et al. [39] when nutrient availability matches or exceeds nutrient requirements.

### (c) Simulations

Genetic differences in the potentials for growth and resistance were incorporated into the model by assigning different values to the parameters *GP* and *ρ* in the equations for growth (1) and immune response (2a), respectively. Thus, a genetically more resistant host, represented by a larger value of *ρ*, would be able to mount a more effective immune response, due to a faster immune cell replication rate (equation 2a) and due to allocating a larger proportion of nutrients towards immunity (equations 3b and 4b).

Simulations were performed for different amounts of available nutrients *N* and different values of the genetic parameters *ρ* and *GP*. For the immunological parameters *α*, *Φ*, *γ*, *λ* and *κ* (equations 2a & b) the scaled estimates from the original model of Antia et al. [39] were adopted, whilst for parameters related to nutrient intake and utilisation (*e_G_*, *e_I_*, *r_min_*) the empirical estimates from Sandberg et al. [43], [44] were used. Sensitivity analysis was performed by modifying one parameter at a time or a combination of parameters within biological reasonable limits, and inspecting the impact of these changes on the model results. Nutrient availability (*N*) was varied from severe limitation (*N* = 6), where the host was unable to control the pathogen (despite meeting maintenance requirements), to the point at which nutrient availability was sufficient to meet all requirements for growth and immunity in the absence of anorexia (*N* = 20). In the case of severe limitations of available nutrients, within host pathogen load could not be controlled by the host's immune response and it was assumed that the host would eventually die (i.e. if pathogen load exceeded 10^50^). Since the focus of this study were interactive effects of nutrition and host genetics on growth and immune response, only results referring to parameter ranges for *ρ* and *GP* corresponding to host survival for both nutritional extremes are presented.

Infection starts with the host being infected with a single micro-parasite, which then replicates within the host. It was assumed that clearance of the pathogen occurs when *P* = 1 is achieved and that re-infection does not take place over the course of the simulations which cover the time period required for clearing the pathogen or reaching the steady state. The duration of the infection was hence defined as the length of time until pathogen load has decreased to unity or reached the steady state.

## Results

### (a) Dynamic trends for pathogen load, immune response and growth

**Figure 1:**
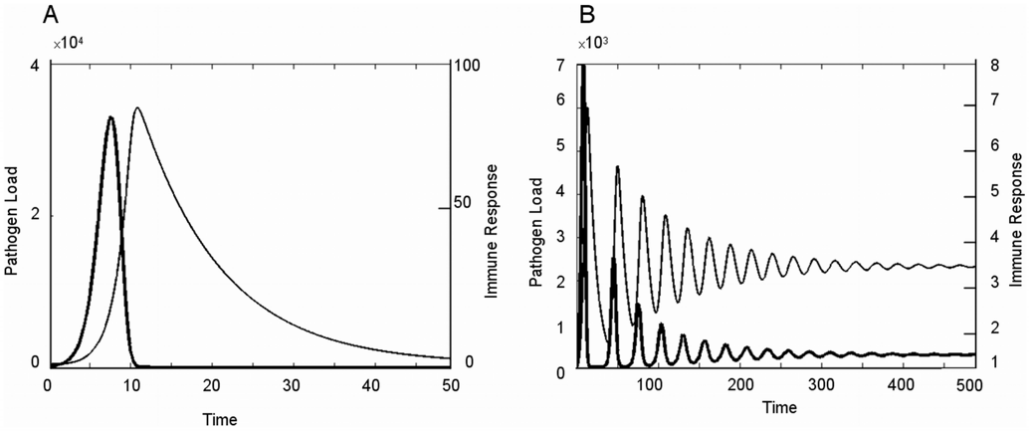
Predicted pathogen load (thick line) and immune response (thinner line) over time. Parameter values used for graph A were *e_I_* and *e_G_* = 0.25, *ν* = 0.01, *r*_min_ = 0.3, λ = 1, κ = 0.05, α = γ = 0.1, Φ = 1000, ρ = 1, GP = 10, N = 8; for graph B the same parameter values were used except for κ, which was set to 0.5 The initial size of the immune response (*I_0_*) and Pathogen Load (*P_0_*) were standardized to unity. For explanation of the parameters see text. All units are arbitrary.

Provided that an infection can be established, the modelled pathogen load and host immune response exhibit one of the following three dynamic patterns: (i) the host immune response clears the infection (Figure 1A), (ii) pathogen load and immune response exhibit damped oscillations towards a persistent steady state with low pathogen load (Figure 1B), or (iii) the host immune response is too weak to control the pathogens and pathogen load increases until death of the host. As mentioned above, only results referring to the first two scenarios are of interest here. The immune response generally lags behind the pathogen load and is characterised by a slower decrease compared to that observed for pathogen load. Even in cases resulting in the eventual pathogen clearance, the immune response persists for an extended period of time (Figure 1A). This behaviour is observed for a wide range of model parameters. Only low values for the resistance parameters *ρ* or extremely low levels of nutrient intake, caused for example by high levels of anorexia (i.e. low values for *r_min_*) or low values of *N* will result in host death. Compared to pathogen clearance, damped oscillations occur if the pathogen elimination rate by the host immune response *κ* is relatively large compared to the pathogen replication rate *λ*. Changes in the model parameter values of *N*, *ρ* and *GP* within the admissible range (i.e. leading to the eventual clearance of pathogens by the host or low persistent pathogen load) alters the time scales of the response curves without affecting the overall shape characteristics of the pathogen load and immune response curves.

Predicted growth rates are piecewise linear (or close to linear), with reduced growth rates during the periods of positive pathogen load.

### (b) Effect of host genetic potentials × nutrition on infection severity and duration

**Figure 2:**
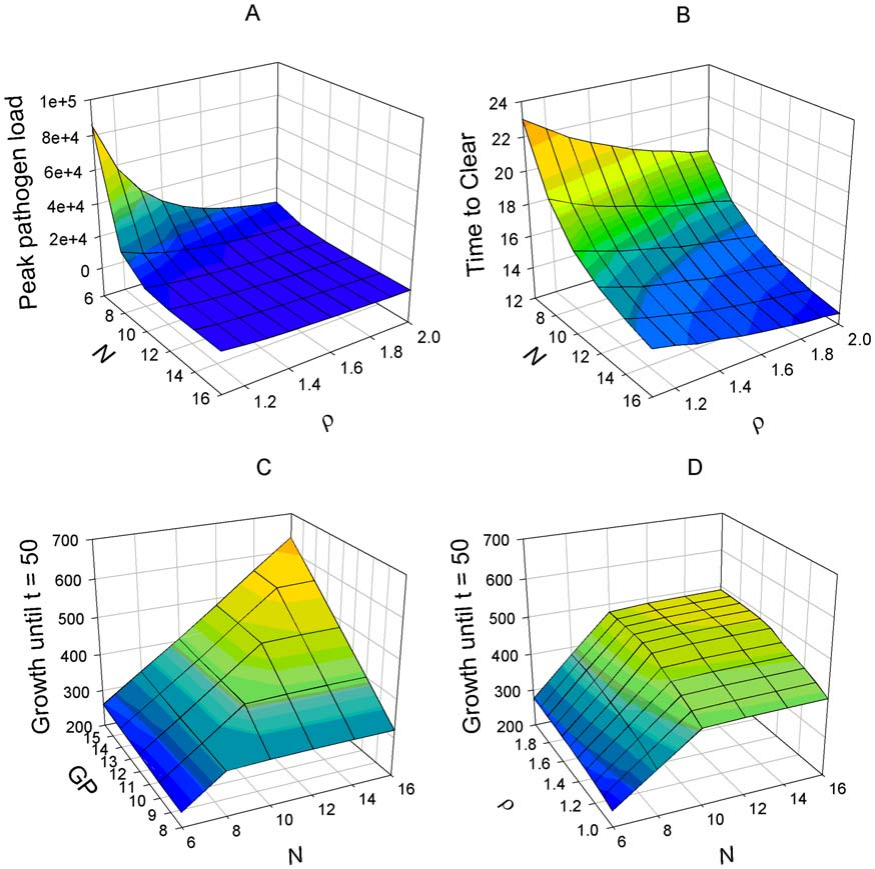
Effects of nutrient availability (*N*) and genetic potential for disease resistance (ρ) and growth (*GP*) on infection characteristics. Graph A: peak pathogen load over time course of infection as function of *N* and *ρ*; graph B: time to clear pathogens as function of *N* and *ρ*. Graphs C and D: and growth until t = 50 as function of *N* and *GP* and *N* and *ρ*, resepectively. The growth potential in graphs A, B & D was *GP* = 10, the resistance potential in graph C was *ρ* = 1.5. For all parameter combinations depicted the infection was cleared at t = 50. Values of other parameters are as in Figure 1. All units are arbitrary. Notice that the rotation angles differ between the individual graphs as they have been chosen for illustration clarity.

**Figure 3:**
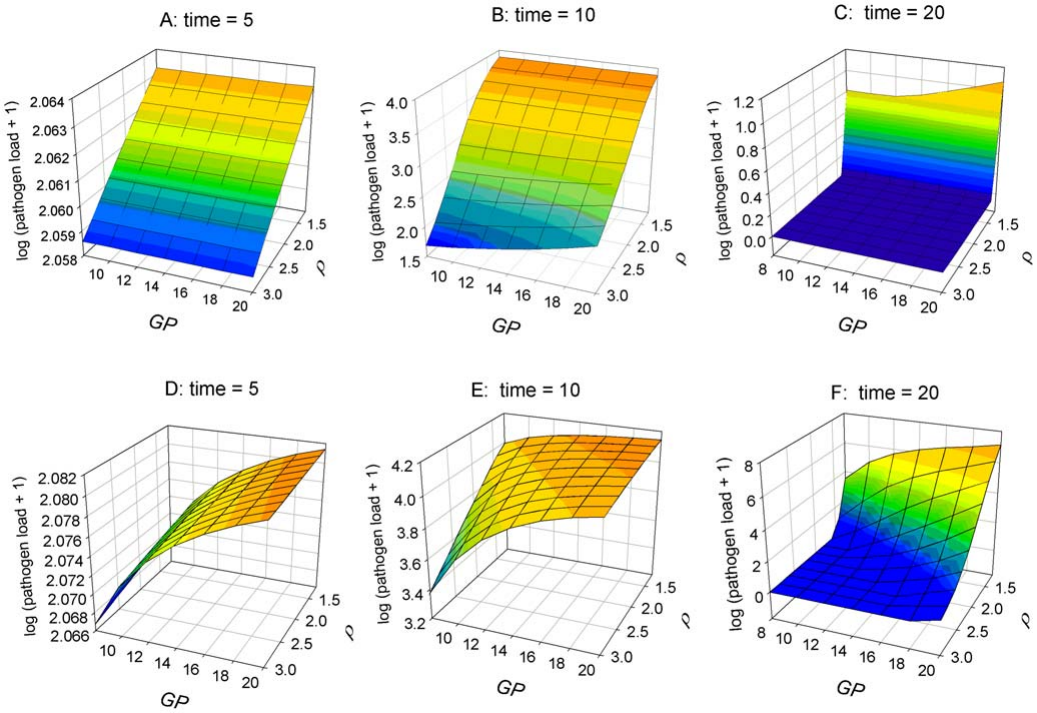
Effect of growth potential (*GP*) and resistance (ρ) on (log_10_ transformed) pathogen load at various times during infection. Graphs A–C: High nutrient availability (*N* = 20); graphs D–F: Low nutrient availability (*N* = 6). Values of other parameters are as in Figure 1. All units are arbitrary. Note that the value ranges for log (pathogen load+1) differ amongst individual graphs, as the value ranges have been chosen for illustration clarity.

The combined effect of host genetic potentials and nutrient availability (N) on infection characteristics and on the predicted pathogen load (log scale) at different time points during the course of infection are shown in Figures 2A & 2B and Figure 3, respectively.

The model predicts that higher amounts of available nutrients generally enable a more effective immune response, resulting in lower pathogen loads at any stage of the infection and in a shorter duration of the infection (Figures 2 & 3). Simulations with gradual variations in nutrient availability and fixed combinations of *GP* or *ρ* revealed that the responses in severity (i.e. peak or cumulative pathogen load) and duration of infection show a diminishing trend: the beneficial influence of increasing nutrient availability on infection characteristics is strongest when nutrient availability is low.

The effect of altering nutrient availability also depends strongly on the host genetic potential. For example, as illustrated in Figures 2A & 2B, increasing nutrient availability is more beneficial for hosts with low genetic resistance potential.

Nutrient availability is predicted to have a strong influence on how growth and resistance potentials interact during the time course of infection. When nutrient availability is high, the within host pathogen load is independent of the growth potential at the early stages of infection, and also hardly depends on it at the later stages (Figures 3A–C). Infection severity and duration are primarily controlled by the resistance potential (*ρ*). The response surfaces of predicted pathogen load for hosts with different growth and resistance potentials change drastically when nutrient supply is limited (Figures 3D–F). In this case, the predicted pathogen load depends more on the genetic potential for growth than for resistance at the early and medium stages post infection (Figures 3D & E). Hosts with lower genetic growth potential are predicted to allocate a higher proportion of available nutrients towards the immune response, thus restricting the replication of pathogens within the host already during the early stages of the infection. The resistance potential has a significant, beneficial influence on pathogen load only when combined with a low growth potential (Figure 3E). However, the influence of the genetic resistance potential increases as time progresses: whereas a resistant host will have managed to clear all pathogens after a certain amount of time (e.g. t = 20 in Figure 3F), pathogen load in susceptible hosts may continue to increase towards very high levels. Low resistance potentials are particularly detrimental for the late stage severity and duration of infection for hosts with simultaneously high growth potentials (Figure 3F).

### (c) Effect of host genetic potential × nutrition on growth under pathogen challenge

**Figure 4:**
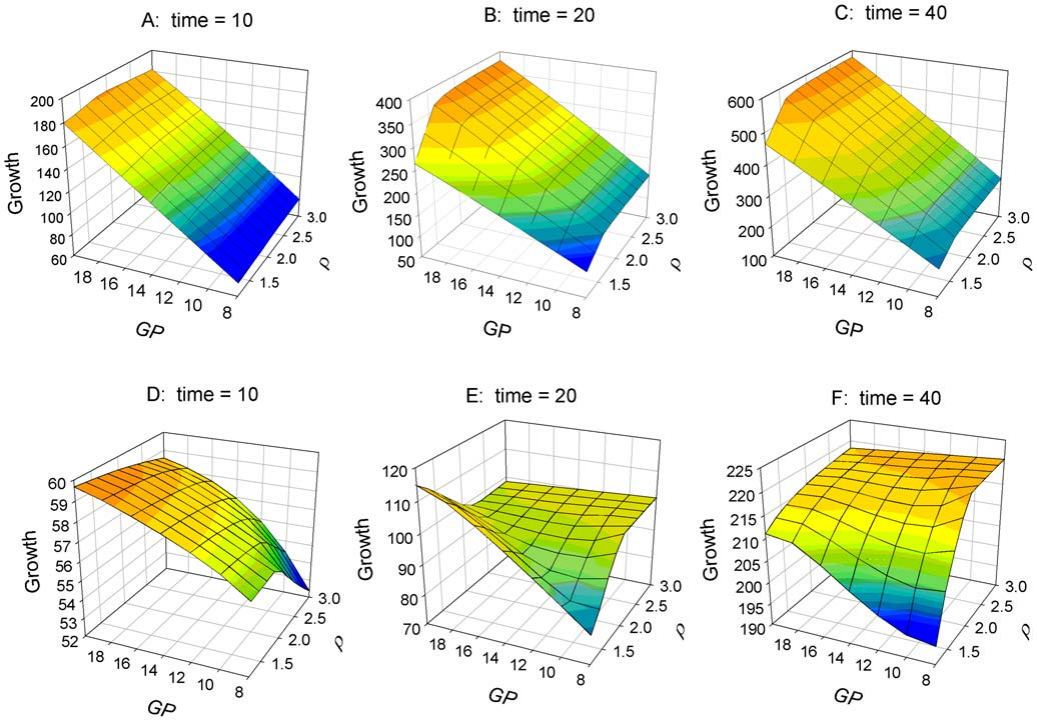
Effect of growth potential (*GP*) and resistance (ρ) on body weight growth at various times during infection. Graphs A–C: High nutrient availability (*N* = 20); graphs D–F: Low nutrient availability (*N* = 6). Values of other parameters are as in Figure 1. All units are arbitrary. Note that the value ranges for growth differ amongst individual graphs and that the rotation angle differs from that of Figure 3, as the value ranges and rotation angles have been chosen for illustration clarity.

The model predicts that higher nutrient availability is beneficial for growth under pathogen challenge for any parameter combinations of *ρ* and *GP* (Figures 2C & D, Figure 4). When considered over a time period that spans the entire infection period, the response to an increase in nutrient availability is similar in infected and non-infected hosts: growth rate increases linearly with increasing nutrients (N) up to a plateau when the amount of available nutrients exceeds the levels needed for satisfying the growth potential (Figure 2C & D). Hosts with higher genetic growth potential reach this plateau at higher levels of N than hosts with lower growth potential and hence benefit from an increase of nutrient availability for a wider range of N values (Figure 2C). Similarly, genetically more resistant hosts benefit from increases in nutrient availability for a wider range of N, although the differences are less pronounced than for hosts with different growth potentials (Figure 2D). The impact of nutrient availability and host genetic potential on achieved growth generally increases over time (Figure 4).

The simulations further reveal that nutrient availability has a strong influence how the genetic potentials for growth and resistance interact towards the actual growth achieved by a challenged host. The model predicts that genetic growth potential is the main driver for achieved growth of infected hosts when nutrient availability (N) is high (Figures 4A–C). Only resistance levels below a certain threshold, which depends on N, produce substantial reductions in growth. These are the consequence of prolonged time periods of ‘severe’ anorexia produced by high pathogen loads over extended time periods.

In contrast, when nutrient availability is low the genetic growth potential is no longer the main driver of achieved growth. Instead, achieved growth depends on the combination of both genetic potentials (Figures 4 D–F). Also, in contrast to the relatively time stable sensitivity surfaces of achieved growth associated with high N (Figures 4A–C), the degree and direction of influence of the growth and resistance potentials change over time when nutrient availability is low (Figures 4D–F). Whereas the model predicts that high growth combined with low resistance potentials are preferable for growth at the earlier stages of infection (Figure 4D & E), low growth combined with high resistance potential appears to be the optimal strategy for achieved growth over the long-term (Figure 4F).

## Discussion

The thesis put forward in this paper was that the conflicting outcomes reported in the literature on the consequence of genetic selection for either host growth or resistance to pathogens may arise from the modification of the interrelationship between these genetic traits by the nutritional environment of the host. Our mathematical model aimed to shed some light on this conflict. The predictions of our model suggest that at higher planes of nutrition improvement in either of these two traits (e.g. by genetic selection) leads to a more effective immune response (seen as resulting low pathogen loads) and higher growth rates respectively, when hosts are exposed to micro-parasites. Thus infection characteristics are primarily determined by the genetic resistance potential (Figures 3A–C) and growth performance is mainly determined by the genetic potential for growth (Figures 4A–C), as would normally be expected [45], [46].

However, the outcome of improvement of exactly the same traits would be very different when the nutritional environment of the host is scarce. In these instances, growth performance and infection characteristics are influenced by both genetic potentials simultaneously, and the influence of either potential depends on the value of the other and varies over time (Figures 3D–F, 4D–F). In particular, the model predicts that an increase of the genetic growth potential would be detrimental to both actual growth and the ability to cope with pathogens when nutrient supply is scarce. This is consistent with the suggestions made by Rauw [27] that selection for production traits, such as growth may negatively influence the ability of animals to cope with pathogens. Our model emphasises that this would be the case, however, only under nutrient scarce environments.

Similarly, according to the model, a high resistance potential in nutrient scarce environments would be detrimental to growth and, if combined with a high growth potential, have no impact on the within-host pathogen load in the short term. Its beneficial effects on both growth and the animal's infectious state depend on the growth potential and may only become apparent over the long term. These predictions can account for previous contradictions in the literature regarding the outcome of selections for disease resistance on actual growth. In farm animal populations, where nutrient availability may be high, selection for resistance would be expected to lead to positive consequences on growth as previously observed [29]. The opposite suggestions about the trade offs between growth and resistance to pathogens made in the ecological literature may arise from the fact that wild animal populations are usually subjected to low or fluctuating nutrient availability.

The model results provide some early insights about the consequences of selection based on phenotypic information upon underlying genetic parameters. The results presented in Figures 3 & 4 imply that the same selection criterion applied in different (nutritional) environments or at different time points may lead to different genetic improvements. For example, in environments with high nutrient availability, selection for low severity and short duration of infection would be equivalent to selecting for high genetic resistance potential, ρ, with no selection pressure acting on the genetic growth potential (Figure 3A–C). Selection for high tolerance to infection in terms of growth in a nutrient rich environment would be equivalent to selecting for high *GP* with little selection pressure on *ρ* (Figure 4A–C). In particular, selection for both observed resistance and tolerance to micro-parasitic infections would be feasible if nutrient availability was sufficiently high (select hosts with simultaneously high *ρ* and *GP*).

In contrast, if nutrients are scarce, selection for low severity and short duration of infection would still favour animals with high genetic resistance potential ρ, but would simultaneously imply selection for low *GP* as only animals with low *GP* allocate the required amount of resources towards immunity (Figure 3D–F). Simultaneous selection for low severity and short duration of infection and tolerance (little impact of infection on growth) would only be possible if tolerance were evaluated over a sufficiently long time period, since in the shorter term, high tolerance implies high *GP* (Figure 4D–F).

The model results also demonstrate the importance of evaluating phenotypic traits over appropriate time periods. For example, although an increase in the genetic resistance potential may produce temporary reductions in growth due to increased investment in the immune response at the early infection stage, growth will benefit in the long-term due to infection having a shorter duration of, and less being less severe (Figures 4D–F). These observations thus match a previous hypothesis that ‘increased investment in immune response in the earlier stages of infection may serve to limit the total resource cost of infection’ [12].

The key drivers determining how host genetic growth and resistance potential and nutrition influence growth and infection characteristics in our model are the pathogen induced anorexia which triggers a conflict between growth and immunity and the allocation of scarce resources towards growth and immune functions.

In our model the reduction of food intake was assumed to depend linearly only on pathogen load up to a minimum level of intake. As a consequence of this assumption, growth and immunity compete for scarce resources only during the time period in which the host is infected with pathogens. The assumption was based on various challenge studies, which showed that food intake reduces with increasing pathogen load until a minimum level is reached that is similar for different levels of infectious dose of micro-parasites [42], [43] and that food intake recovers almost instantaneously when pathogens are removed artificially (eg through drug administration) [47]. Alternatively, given that anorexia is frequently considered as part of the host immune response [20], [22], anorexia could also have been represented as being a function of the host immune response. Our sensitivity analysis indicated that the influence of different anorexia rules (e.g. partial to full dependence on the immune response) and of different values for the parameters *ν* and *r_min_* is generally low compared to the relative influence of the growth and resistance potentials under given nutrient availability. In particular, more severe or more prolonged anorexia (produced e.g. by assuming partial dependence on the immune response) would cause a trade-off between growth and immune response for higher levels of nutrient availability and thus increase the relative influence of the genetic potentials on observed growth and infection characteristics.

The relative impact of the genetic potentials for growth and resistance on predicted growth and infection characteristics strongly depends on the way that available nutrients are allocated, which we assume to be relative to the nutrient requirements for either process (equations 4a, b). Substantial differences exist between the nutrient allocation rules adopted in different modelling studies [3], [19], [34]. The rule chosen here implies that resource allocation partly depends on the host genetic potential for growth and disease resistance and varies over time according to the physical state of the animal [19], [48]. Also, the immune response is not given absolute priority for nutrients. Evidence in support of the latter is provided by experiments which have observed simultaneous increases in growth and expression of an immune response, in response to increased ‘resource intake’ [49]–[51]. Medley [3] proposed a model to assess the optimum allocation of resources for different resource availabilities assuming constant exposure to parasites throughout a host's lifetime. He assumed that the immune response has total priority over other processes (growth and reproduction) for resources and that the proportion of resources allocated to the immune response depends on the pathogen load and does not exceed a fixed proportion of the total amount of resources available to the host. This led to the conclusion that individuals on low nutritional planes should put increasingly less resources into immunity [3]. Our model also predicts that the influence of the genetic potential for resistance on infection characteristics may be reduced as nutrient availability decreases, but this influence also depends on the animal's genetic growth potential.

Other studies have assumed that nutrient allocation is largely controlled by the host genotype [23], [25]. The allocation rule adopted here indirectly implies host genetic influence on the distribution of resources, as a host with higher growth potentials but similar resistance potential relative to another host will allocate a higher proportion of resources towards growth than the immune response under the same pathogen challenge. The role of direct genetic effects on nutrient allocation (i.e. hosts with similar genetic potentials for growth and resistance have different genetically determined preferences for allocating nutrients) has been investigated in a previous study [25]. It was found there that compared to the impact of host genetic potentials for growth and resistance, genetically determined nutrient allocation had a relatively small impact on observed growth and immune response.

A simple approach was adopted here to obtain insight into the interacting effects of host genetic potentials and nutrition on growth and infection characteristics without adding complexity caused by other contributing factors (e.g. other environmental stressors or other biological processes such as reproduction). This approach allowed new interpretations of the apparently ambiguous outcomes reported in selection studies. It also revealed the possible side effects of selecting for one or several genetic traits that compete for nutritional resources. The simplistic representation of pathogen challenge, host genetic potentials, nutrition and immunity by single entities makes direct comparison of our model results with real data from challenge or selection studies difficult. The proposed framework could however easily be adapted to include more complex representations of the influencing factors and of the biological processes affected by them (e.g. different arms of immune response, reproduction). The hope is that such models can be then expanded to predict performance of animals under exposure to specific pathogens.
